# Circadian Modulation of Anxiety: A Role for Somatostatin in the Amygdala

**DOI:** 10.1371/journal.pone.0084668

**Published:** 2013-12-20

**Authors:** Anne Albrecht, Marlen Thiere, Jorge Ricardo Bergado-Acosta, Janine Poranzke, Bettina Müller, Oliver Stork

**Affiliations:** 1 Department of Genetics & Molecular Neurobiology, Institute of Biology, Otto-von-Guericke University Magdeburg, Magdeburg, Germany; 2 Center for Behavioural Brain Science, Magdeburg, Germany; Max Planck Institute of Psychiatry, Germany

## Abstract

Pharmacological evidence suggests that the neuropeptide somatostatin (SST) exerts anxiolytic action via the amygdala, but findings concerning the putative role of endogenous SST in the regulation of emotional responses are contradictory. We hypothesized that an endogenous regulation of SST expression over the course of the day may determine its function and tested both SST gene expression and the behavior of SST knock out (SST^-/-^) mice in different aversive tests in relation to circadian rhythm. In an open field and a light/dark avoidance test, SST^-/-^ mice showed significant hyperactivity and anxiety-like behavior during the second, but not during the first half of the active phase, failing to show the circadian modulation of behavior that was evident in their wild type littermates. Behavioral differences occurred independently of changes of intrinsically motivated activity in the home cage. A circadian regulation of SST mRNA and protein expression that was evident in the basolateral complex of the amygdala of wild type mice may provide a neuronal substrate for the observed behavior. However, fear memory towards auditory cue or the conditioning context displayed neither a time- nor genotype-dependent modulation. Together this indicates that SST, in a circadian manner and putatively via its regulation of expression in the amygdala, modulates behavior responding to mildly aversive conditions in mice.

## Introduction

In recent years, the importance of neuropeptides for the pathogenesis of anxiety disorders has increasingly been recognized and has led to their consideration as potential therapeutic targets [1,2]. Evidence suggests that somatostatin (SST) is one neuropeptide with potent anxiolytic properties [3,4] and a role in fear memory formation [5], which may thus hold significant potential for the treatment of anxiety- and stress-related disorders [6]. 

The anxiolytic activity of SST appears to be exerted at least in part via the amygdala [3,4], where it is endogenously expressed in a subpopulation of GABAergic interneurons with defined morphological and electrophysiological properties [7,8]. These interneurons form local microcircuits with the principal cells that are critically involved in the processing of fear- and anxiety-related information [9]. SST itself contributes to these functions by inhibiting principle cells mainly via the type 2 SST receptor coupled to inwardly rectifying calcium channels [10].

However, no or only modest effects of SST null mutation have been so far observed on amygdala-dependent functions [5,11]. We hypothesized that the reason for this may involve expression regulation of endogenous SST, such as the daytime of testing, and that the phenotype of SST null mutants may depend these testing conditions. In fact, circadian fluctuation of gene expression has been repetitively reported in fear circuits [12–14] and for anxiety- and fear-related behavior [15,16]. 

We therefore investigated anxiety-like and conditioned fear behavior of SST^-/-^ mice in two different periods of the dark cycle, i.e. during their active phase. A further analysis of mRNA and protein expression revealed corresponding changes of SST expression in the basolateral amygdala (BLA) and its co-modulation with its co-transmitter Neuropeptide Y (NPY) and the clock gene Period 2 (Per2). Our data suggest a role of amygdalar SST in the circadian modulation of anxiety levels under moderately aversive conditions.

## Materials and Methods

### Circadian behavior of SST deficient mice

#### Animals

All studies were conducted in accordance with the European and German regulations for animal experiments and were approved by the Landesverwaltungsamt Sachsonia-Anhalt (AZ 42502/2–441 UniMD). SST deficient mice (SST^-/-^; backcrossed to C57Bl/6 for >12 generations) were obtained from heterozygous breeding and raised in groups of 2-6 under standard laboratory conditions with a 12h day/night cycle (lights off at 7:00 a.m. with 30 min dawn phase) and food and water ad libitum. Genotypes were determined by multiplex polymerase chain reaction on genomic DNA, as described previously [5]. Mice were separated one week before the experiments and housed individually throughout the behavioral assessment. Training and testing of animals were always done either in the first half of the dark cycle, starting 1h after the beginning of the dark phase (D1^st^; 8.00 - 10.00 a.m.) or in the second half of the dark phase, starting 7h after the beginning of the dark phase (D2^nd^; 14.00- 16.00 p.m.). 

Adult male SST^-/-^ and SST^+/+^ mice underwent a battery of tests to assess their activity patterns in the home cage, anxiety like behavior in the open field and the light-dark-avoidance test and auditory cued fear conditioning. 

#### Home cage activity

Single-housed SST^-/-^ (N=12) and SST^+/+^ mice (N=12) were left undisturbed in their home cages for 3 days while infrared sensor collocated on top of our standard wire home cage cover, providing x, y, and z axis coverage, measured their activity. The sensor head consisted of infrared motion sensors mounted on top of the cage (Home Cage Activity System, Coulbourn Instruments, Allentown PA). Raw values 15 s of activity were collected at 0.01Hz and used to detect activity periods in 5 min bins. The proportion of active and inactive periods per hour was determined as a three-day average in a 24 h-cycle (% activity). In addition average activity over the time period of behavioral testing at the first (D1^st^ 8.00 - 10.00 a.m.) versus the second half of the dark phase (D2^nd^; 14.00- 16.00 p.m.) was determined. 

#### Open field

Mice were placed in the center of a square arena made out of grey plastic (50 cm x 50 cm) and were allowed to explore the arena freely for 20 min at low light conditions (10 lux). Animal’s behavior was recorded online via the ANYMAZE video tracking system. The distance covered during the test session was analyzed as a parameter for general activity, the time spent in the center of the open field (a rectangle 20 cm from sidewalls) was determined to assess anxiety levels. Mice of both genotypes were randomly assigned to two groups either receiving the open field test in the beginning of the dark phase (D1^st^; N for SST^+/+^ = 6; N for SST^-/-^ = 7) or in the second half of the dark phase (D2^nd^; N for SST^+/+^ = 8; N for SST^-/-^ = 7). Due to technical problems in automated behavioral tracking one animal was excluded from analysis. 

#### Light- Dark- Avoidance Test

One day after the open field tests, animals tested in the open field at D1st received the light-dark-avoidance test now at D2^nd^ and vice versa. All Animals were placed in the light compartment (100 lux, 19 cm x 21 cm) of the testing chamber (TSE, Bad Homburg, Germany). The brightly illuminated compartment was joined with a dim dark compartment (< 1 lux, 16.5 cm x 21 cm) by an opening (3.7 cm x 4 cm) in the wall’s bottom center. During the five minutes session all mice were allowed to explore both compartments freely. Animal’s locomotion was detected online by a photo beam activity system. Distance walked and time spent in light compartment indicated decreased anxiety levels. Due to technical problems in automated behavioral tracking one animal was excluded from analysis (D1^st^: N for SST^+/+^ = 8; N for SST^-/-^ = 6; D2^nd^: N for SST^+/+^ = 7; N for SST^-/-^ = 7).

#### Auditory cued fear conditioning

All training and test sessions took place in a sound isolation cubicle containing a 16 cm x 32 cm x 20 cm acrylic glass arena with a grid floor, loudspeaker and ventilation fan (background noise 70 dB SLP, light intensity <10 lux; TSE, Bad Homburg, Germany). Prior to fear conditioning, all animals received four (twice per day, i.e. morning and afternoon) adaptation sessions consisting of 2 min exposure to the conditioning context, followed by six exposures to a neutral tone (CS-: 2.5 kHz for 10 s, 80 dB) with 20 s interstimulus intervals (ISI) between each tone exposure. On the consecutive day paired auditory cued fear conditioning training took place. Here, animals of both genotypes were randomly assigned to two groups, receiving the training either at D1^st^ (N for SST^+/+^ = 7; N for SST^-/-^ = 7) or at D2^nd^ (N for SST^+/+^ = 8; N for SST^-/-^ = 7). After 2 min exposure to the training context, animals received three footshocks (US: 0.4 mA for 1s), each paired with a tone (CS+: 10 kHz for 10 s, 80 dB) and separated by 20 s ISI. Two days later fear memory to the auditory cued tone and the training context was tested separately in two retrieval sessions for all animals at D1^st^. For retrieval of the auditory cue mice were placed a neutral context resembled by a plexiglas standard cage with bedding placed in the cubicle of the fear conditioning apparatus. After 2 min in the neutral context, the animals were re-exposed to 4 CS- (10 s each, 20 s ISI) and 4 CS+ (10 s each, 20 s ISI). One hour after the cue retrieval, the test animal was re-exposed to the training context for 2 min. The animal’s behavior during both sessions was assessed online via a photobeam detection system that detected immobility periods < 1 s and activity bursts (< 20 cm/s). As shown previously, the automatically gained immobility periods correlate well with observer rated freezing behavior [17].

#### Statistical analysis

Home cage activity was analyzed using Repeated measures ANOVA over 24 h with genotype as between subject factor. Average activity at the two time phases of behavioral testing, D1^st^ and D2^nd^, was compared between genotypes using Student’s t-test. Effects of genotype and time phase of testing on anxiety-like behavior and fear memory were determined with a multiple analysis of variance (MANOVA), followed by Fisher’s PLSD for post hoc comparison when necessary. Planned comparisons were conducted to specify genotype or time-of-testing effects within respective groups. 

### Circadian gene expression in the BLA

#### Animals

All studies were conducted in accordance with the European and German regulations for animal experiments and were approved by the Landesverwaltungsamt Sachsonia-Anhalt (AZ 42502/2–441 UniMD). Adult male C57B/6BomTac (M&B Taconic, Berlin, Germany) were obtained at an age of seven weeks and housed in our animal facility for two weeks in a 12 h light/dark cycle with lights off at 7:00 a.m. and food and water ad libitum. After one week of single housing animals (N=4 per time point) were sacrificed at different time point of the 12 h light/ 12 h dark cycle starting 1 h after lights off (T1) and then every three hours (T4= 4 h after light off; T7= 7 h after lights off; T10= 10 h after lights off; T13= 1 h after lights on; T16= 4 h after lights on; T19= 7 h after lights on; T22= 10 h after lights on). 

#### RNA preparation from Amygdala tissue and reverse transcription

After brief narcosis with isofluran mice were sacrificed, their brains were isolated and cut into 300 µm slices on a slicer matrix kept on ice (Zivic instruments, Pittsburgh, PA, USA). The basolateral complex of the amygdala (BLA) was isolated via a stainless steel puncher with a diameter of 0.5 mm (Zivic instruments, Pittsburgh, PA, USA). Bilateral BLA punches of each animal were collected in 1.5 ml eppendorf tubes containing 100 µl lysis buffer and incubated for 10 min at 75°C. The tissue was further processed using the “Cells-to-cDNA II”- Kit (Ambion, Huntingdon, UK) according to manufactors instructions. In brief, lysates were treated with DNAse I (0.04 U/µl) for 15 min at 37°C, followed by DNAse inactivation for 5min at 75°C. First-strand synthesis was done with M-MLV reverse transcriptase (100 U/µl) in the presence of 2.5 mM dNTPs, 50 µM oligo-dT primer oligonucleotide and RNAse Inhibitor (10 U/µl) for 60 min at 42°C, followed by enzyme inactivation at 94°C for 10 min.

#### Real time PCR

Quantitative PCR was performed with the StepOne Plus Real-Time PCR system (Applied Biosystems, Darmstadt, Germany) using TaqMan® reagents with predesigned assays for Per2 (Mm00478113_m1), SST (Mm00436671_m1), Gad2 (Mm_0484623_m1), Gad1 (Mm_01271479_m1); CCK (Mm_00436671_m1), NPY (Mm00445771_m1) and the housekeeping gene glyceraldehyde 3-phospahte dehydrogenase (GAPDH; 4352923E) “TaqMan gene expression assays”, Applied Biosystems) in triplicate assays. Labeling of the different target genes and the housekeeping genes with different fluorescence dyes allowed for multiplex PCR using GAPDH as internal reference. Pre-experiments confirmed that GAPDH expression was independent of treatment groups and referred only to the starting amount of DNA in each sample. All runs consisted of 50 cycles with 15s at 95°C and 1 min at 60°C and were preceded by a 2 min 50°C decontamination step with Uracil-N-Glycosidase. 

#### Data analysis

Mean cycle threshold (CT) values were determined for each triplicate assay and used for sample comparison according to the ddCT method [18]. Here, CT values of the different target genes were normalized to GAPDH for each sample (dCT = CT(target gene) – CT(GAPDH)). Expression differences between different time points were analyzed with non-parametric Kruskal-Wallis test based on the dCT values for each sample and target gene. Correlation of mRNA levels for the different neuropeptides as well as GAD65 and GAD67 with the circadian gene Per2 over the different time points was assessed by Pearson’s correlation coefficient. Expression differences between T1 and T7, the starting points for behavioral analysis of SST deficient mice, were determined by Mann-Whitney U test. 

### Circadian expression of SST peptide in the BLA

Punches of the BLA were prepared as described above from male adult C57Bl/6 mice starting either at T1 (1 h after lights off; T1 – T2: 8.00 to 9.00 a.m.; N=7) or at T7 (7 h after lights off; T7 – T8: 14.00 to 15.00 p.m.; N=7). Bilateral BLA punches of each animal were transferred to a tube containing lysis buffer (1 % DDM, 1 % NP40, 1 mM Na3VO4, 2 mM EDTA, 50 mM Tris-HCl (pH 8, 4°C), 150 mM NaCl, 0.5 DOC, 1 mM AEBSF, 1 µM Pepstatin A, 10 % Gycerol (all reagents from Sigma, and 3 tablets of Protease Inhibitor (Thermo Fisher Scientific Inc., Rockford, USA)) and homogenized. Samples were then incubated at 4°C on a rotor platform for 20 min, centrifugated at 1000 x g for 20 min at 4°C and stored at -80°C. Determination of somatostatin peptide expression levels was done via Enzyme-linked Immunosorbent Assay (ELISA) according to manufacturer’s instructions (Uscn Life Science Inc., Wuhan, China) and statistical comparison of the two time points was done by Mann-Whitney U test.

## Results

### Circadian behavior of SST deficient mice

#### Lack of circadian fluctuation of anxiety-like behavior in SST mutant mice

**Figure 1:**
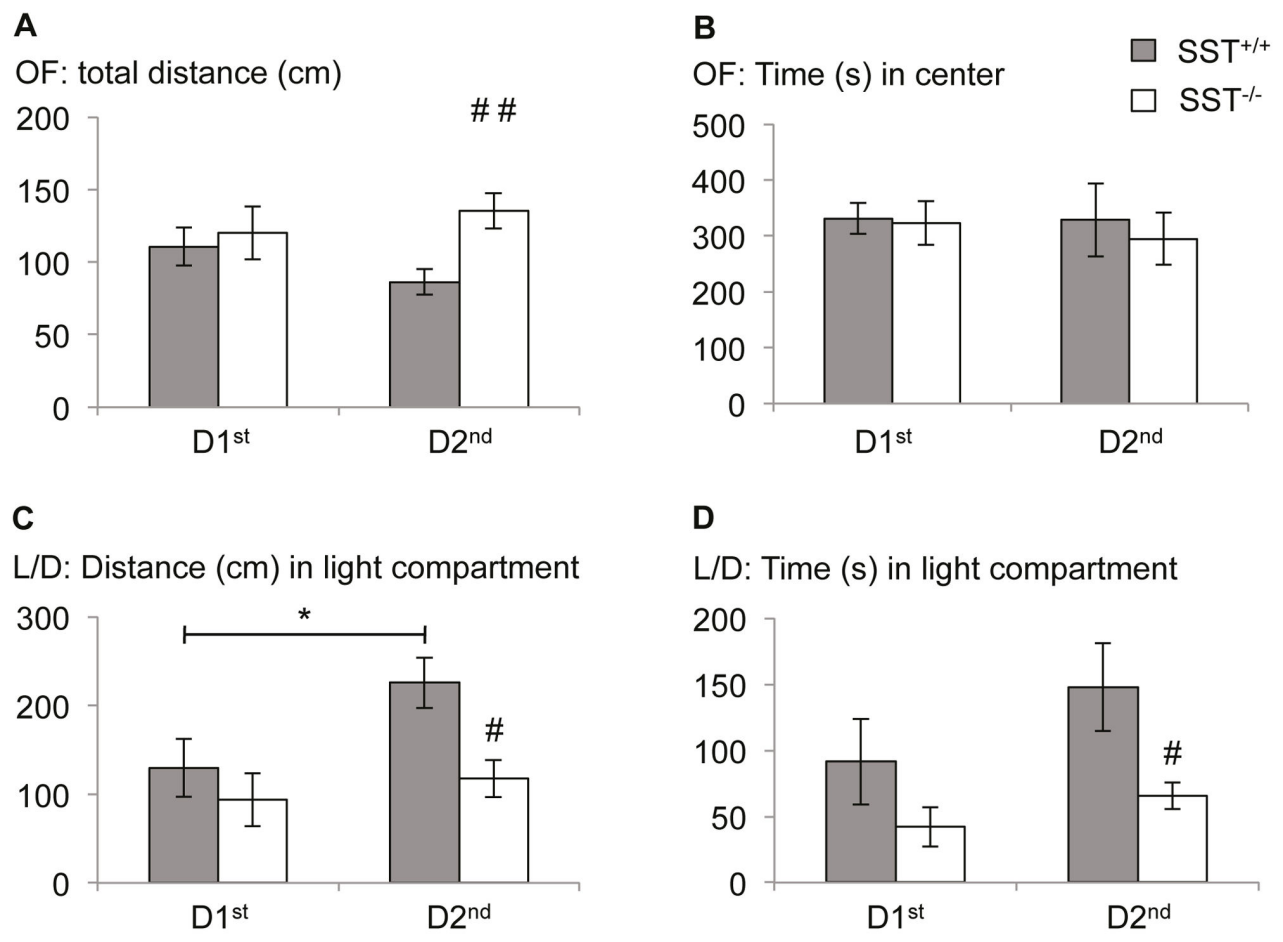
Lack of circadian fluctuation of anxiety-like behavior in SST deficient mice. (A) SST^-/-^ mice displayed hyperactivity during the second half of their active phase as indicated by total distance walked in the open field, compared to their SST+/+ littermates. (B) However, time spent in center did not differ between genotypes are time of testing. (C,D) In the light/dark- avoidance test, SST^-/-^ mice showed reduced exploration of the light compartment, indicating increased anxiety-like behavior during the second half of the active phase. In fact the mutants failed to show a reduction of anxiety as seen in SST^+/+^ mice, thus indicating a deficit in the circadian modulation of anxiety- like behavior. Values are mean ± SEM. * significant differences between time point of testing, p<0.05; #, significant differences between SST^-/-^ and SST^+/+^ mice, p < 0.05; # # p < 0.01.

Multivariate ANOVA for genotype x time of testing revealed significant effects of genotype (F(1,24)=4.792; p=0.039) on general activity of SST^-/-^ mice and their wildtype littermates as indicated by the total distance walked in the 20 min open field test sessions (effect of time: F(1,24)=0.118; p=0.734; interaction genotype x time: F(1,24)=2.159; p=0.155). Distance was slightly increased in SST^-/-^ mice at the 2^nd^ half of the dark phase and slightly decreased in SST^+/+^ mice, resulting in a genotype specific effect at D2^nd^ (Figure 1A; F(1,13)=10.982, p=0.006), but not D1^st^ (F(1,11)=0.172; p=0.686). 

The time spent in the center of the open field (Figure 1B) as a measure for anxiety-like behavior was neither affected by genotype (F(1,24)=0.172; p=0.682) nor time point of testing (F(1,24)=0.100; p=0.755), nor the interaction of both factors (F(1,24)=0.066; p=0.799). 

These data indicates a moderate hyperactivity of SST^-/-^ mice which is more pronounced at D^2nd^. 

In the light-dark-avoidance test, a multivariate ANOVA revealed that the activity of the animals in the light compartment as indicated by the distance walked there was significantly affected by genotype (F(1,24)=6.226, p=0.02) and time of testing (F(1,24)=4.302; p=0.049; interaction genotype x time: F(1,24)=1.563; p=0.223). The distance walked in the light compartment was significantly enhanced at D2^nd^ in SST^+/+^ mice, but not in SST deficient mice, resulting in a genotype specific effect at D2^nd^ (Figure 1C; F(1,12)=9.403, p=0.01), but not D1^st^ (F(1,12)=0.615; p=0.448). 

A similar effect was also observed for the time spent in the light compartment with significant effects of genotype (F(1,24)=6.148, p=0.021; time of testing: F(1,24)=2.271; p=0.145; interaction genotype x time: F(1,24)=0.389; p=0.539). Again, SST^+/+^ spent more time in the light compartment when tested at D2^nd^ than SST^-/-^ mice, resulting in a genotype specific effect at D2^nd^ (Figure 1D; F(1,12)=5.626, p=0.035), but not D1^st^ (F(1,12)=1.502; p=0.244). 

Together, this data indicates a day time- dependent modulation of anxiety- like behavior in wildtype mice with decreased anxiety in the second half of the dark phase. SST^-/-^ mice did not display such a circadian modulation of anxiety-like behavior. 

#### Normal circadian activity patterns in SST mutant mice

**Figure 2:**
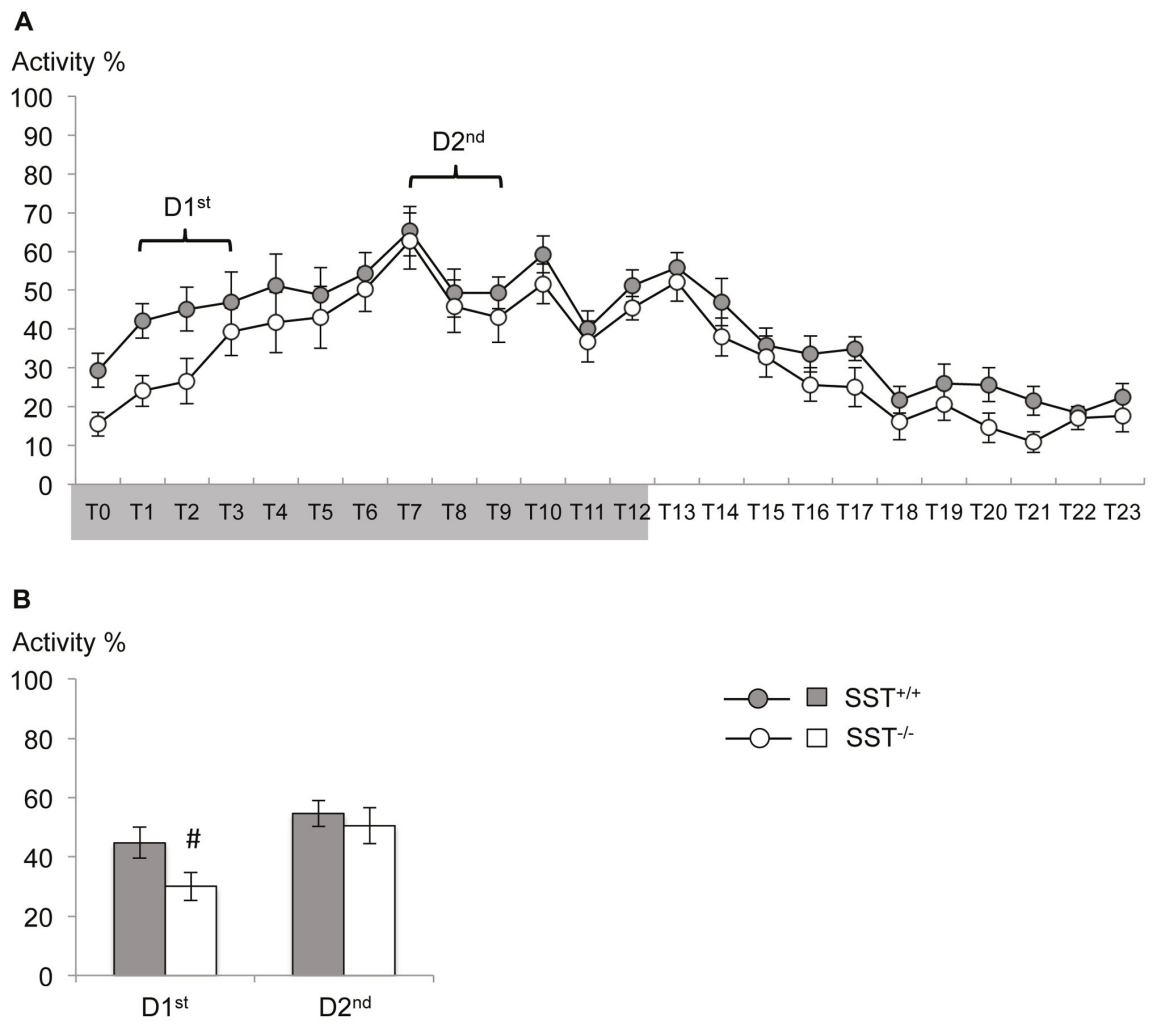
Home cage activity of SST deficient mice. (A) The general activity profile of SST^-/-^ mice in their home cages did not differ from that of SST^+/+^ mice over the 24h cycle (lights off indicated by gray shaded area on the x-axis). (B) Planned comparison of the average activity over the daytime corresponding to the behavioral test periods showed a reduced activity of SST^-/-^ mice during the first half of the active phase (D1^st^) but no difference between genotype in the second half (D2^nd^). Values are mean ± SEM. #, significant differences between SST^-/-^ and SST^+/+^ mice, p < 0.05.

Repeated measure ANOVA indicated a comparable activity patterns between SST^-/-^ and SST^+/+^ mice in their home cages within a 24 h cycle (Figure 2A; time: F(23,506)=21.843; p=0.000; time x genotype: F(23,506)=0.594; p=0.933; genotype: F(1,22)=2.757; p=0.111), with low activity at the beginning of the dark phase that increases towards the second half of the dark phase and declines again over the light, inactive phase of the animals.

However, when analyzing the time phases behavioral testing was done, SST^-/-^ mice were less active at the beginning of the dark phase (D1^st^) compared to their wildtype littermates (Figures 2B; 2-tailed Student’s t-test: T(22)=2.109; p=0.047), while activity was increased during later stages of the dark phase in both genotypes (D2^nd^;T(22)=0.545; p=0.592). 

Although activity patterns appear undisturbed in general, display SST^-/-^ mice a slightly protracted increase of activity levels at the beginning of the dark, active part of the day.

#### No influence of day time of training on auditory cued fear memory

**Figure 3:**
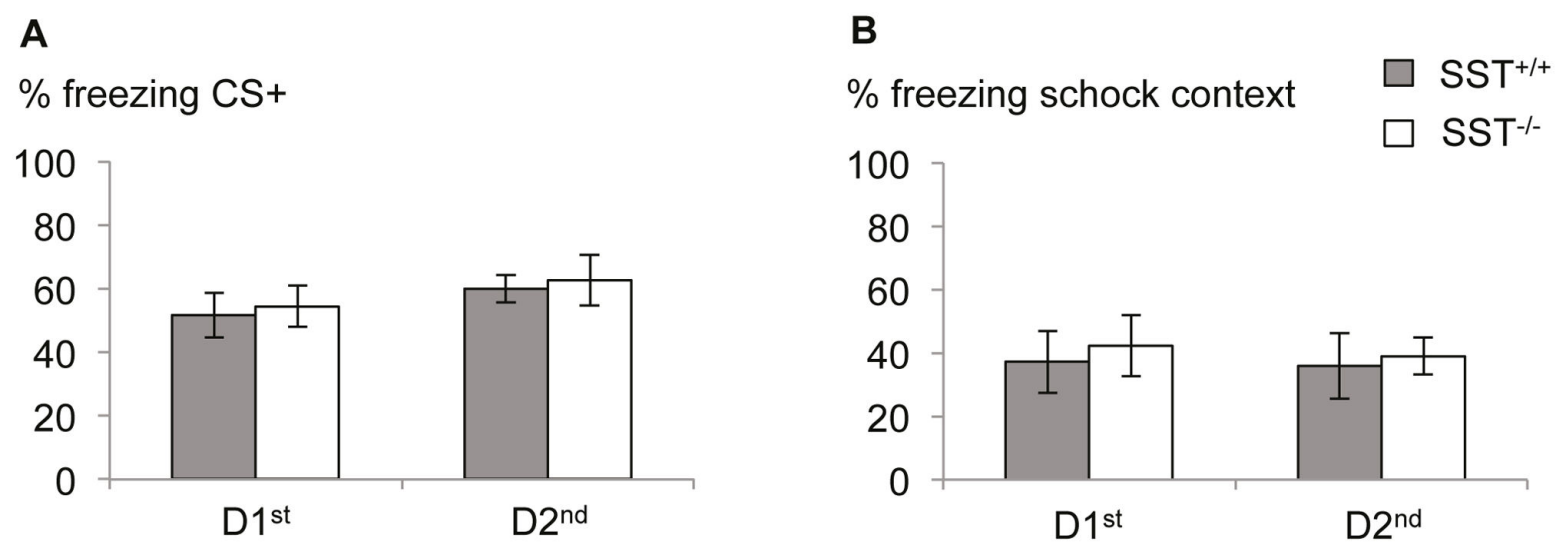
Unaltered auditory cued fear memory in SST deficient mice. (A) Freezing towards the conditioned auditory stimulus (CS+) did not differ between genotypes or time points of testing. (B) Likewise, treezing towards the context also was not influenced by the time point of training. No deficits in could be observed in SST^-/-^ mice. Values are mean ± SEM.

Auditory-cued fear memory was neither significantly affected by the genotype of the animals (F(1,25)=0.174; p=0.68) nor by the time the fear conditioning training took place (F(1,25)=1.663; p=0.209), nor by the interaction of both factor (F1,25)=0,00; p=0.993), as revealed by multivariate ANOVA towards the freezing response during CS+ retrieval (Figure 3A). Also the freezing response towards the context (Figure 3B) in which the tone training has found place did not differ between animals of different genotypes (F(1,25)=0.206; p=0.654) or different training time points (F(1,25)=0.061; p=0.807) or the interaction of both factors (F(1,25)=0.011; p=0.918). Likewise, generalization of the conditioned fear response towards a neutral tone as measured with freezing to the CS- was not observed depending on genotype (F(1,25)=1.507; p=0.231) or training time (F(1,25)=1.116; p=0.301; interaction genotype x time: F(1,25)=0.001; p=0.977). 

These results indicate no circadian regulation of fear memory consolidation. SST^-/-^ mice displayed no deficits in tone or context-dependent fear response. 

### Circadian gene expression in the BLA

**Figure 4:**
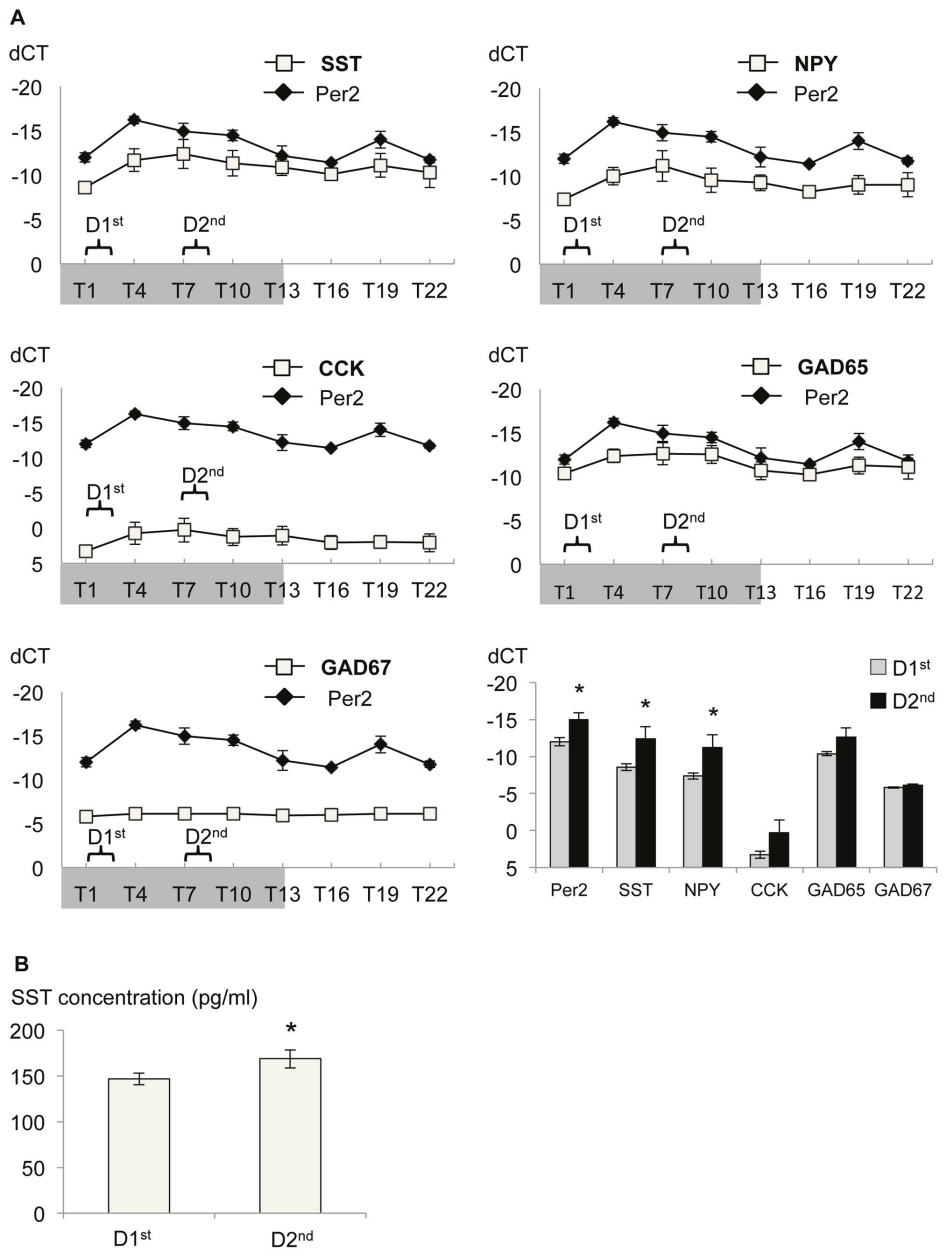
Circadian modulation of SST expression in the BLA. (A) Per2 mRNA levels display pronounced circadian fluctuation over a 24h period, confirming previous reports. Expression of SST is well correlated (Pearson’s correlation: r=0.439) with expression levels of Per2 in the BLA over the different time points. Significant correlation with Per2 expression is also observed for NPY (r=0.455) and GAD65 expression levels (r=0.514). Shaded area illustrates 12h lights off period and brackets indicate time of behavioral testing. Planned comparison revealed a significant increase of Per2 and SST, but also NPY mRNA levels towards the second half of the active phase (D2^nd^). Values are relative expression after normalization to house keeping gene GAPDH (dCT) ± SEM for each time point. (B) Accordingly, the peptide concentration of SST in the BLA is increased in the second half of the dark phase compared to the first half.. Values are mean ± SEM. * significant difference T1 vs. T7, p<0.05 .

Over a 24h cycle, measured every three hours, starting one hour after lights off (T1), Per2 mRNA levels displayed circadian expression differences in the BLA (Figure 4A; Kruskal-Wallis: p=0.016) with lower expression levels at D^1st^ that increase three hours later and are still elevated at D^2nd^ (Mann-Whitney U test: p=0.043). A circadian expression of the clock gene Per2 was well correlated with the expression pattern of GAD65 (2-tailed Pearson’s correlation coefficient r=0.514; p=0.003) and the neuropeptides SST (r=0.439; p=0.013) and NPY (r=0.455; p=0.010), but not with GAD67 (r=0.327; p=0.072) and CCK (r=0.339; p=0.062) expression patterns in the BLA. 

The low mRNA level at the beginning of the dark phase (D1^st^), that increased towards the second half of the dark phase (D2^nd^) observed for Per2 was also seen for SST (Figure 4A; Mann-Whitney U test: p=0.043) and NPY (p=0.043), suggesting a circadian modulation of SST and NPY mRNA levels in the BLA with dynamic modulation during the dark phase.

### Circadian expression of SST peptide in the BLA

As demonstrated via ELISA, did not only SST mRNA levels but also SST peptide levels display a small, but significant increase from the beginning (D1^st^) towards the second half of the dark phase (D2^nd^) in the BLA (Figure 4B; Mann-Whitney U test: p=0.048).

## Discussion

Circadian modulation of fear and anxiety can be observed in generalized anxiety disorder and panic patients [19,20]. It also strongly determines the outcome of behavioral tests in animal models of anxiety disorders [15,21]. In this study, we identified SST as a mediator of such circadian regulation of anxiety-like behavior in mice and describe the corresponding modulation of SST expression within the basolateral amygdala.

To obtain insight into the circadian regulation of activity, fear and anxiety, we studied SST^-/-^ mice during two time periods of their active phase, i.e. in the first or the second half of the dark phase of the light / dark cycle, using a set of tests with different levels of averseness. Care was taken that the least aversive tests were performed first to minimize potentially confounding effects of previous stress experience on test performance. In an open field test, activity was enhanced in SST^-/-^ mice in a daytime-dependent manner, as the mutants but not their wildtype littermates increased locomotor activity towards the second half of the dark phase. In the more aversive light/dark- test, we did not detect differences in parameters of general activity between genotypes or time points, but found a reduced anxiety-like behavior of SST^+/+^ mice during the second half of the dark phase, confirming our recent observations in C57Bl6 mice [22]. However, no such circadian change was observed in SST^-/-^ mice. As the assessment of home cage activity revealed not genotype effect in this time period, these differences are likely to reflect a deficit of SST^-/-^ mice in the circadian modulation of anxiety-like behavior. Intrinsic reduction of home cage activity in SST^-/-^ mice during the first half of the dark phase, on the other hand, was not reflected in altered anxiety levels.

Pharmacological experiments have revealed anxiolytic activity of SST after microinjections into ventricles, amygdala or septum [3,4]. In a previous study, SST deficient mice displayed only insignificant trends towards increased anxiety in the light/ dark- avoidance test, but details about time points of testing were not provided [11]. Together with our current data, it is thus plausible that increased utilization of endogenous SST may provide anxiolytic-like effects. Indeed, we observed increased expression of SST mRNA and protein levels in the basolateral amygdala of C57Bl/6 wild type mice during the second half, compared to the first half of the dark period. Increased SST in the BLA may exert anxiolytic effects via activation of the SST type 2 receptor (SST R2) [3,4] and could in fact explain both the reduced anxiety-like behavior of SST^+/+^ mice and the failure of SST^-/-^ mice to show this change during the second half of the active phase. 

The basolateral amygdala is critically involved in the control of anxiety and the formation of fear memories in humans and rodents [23,24]. However, we could not observe any effect of genotype on fear memory towards the auditory cue or the training context. This is in line with previous observation with high US intensities (0.7mA [11]), but differs from the disturbed acquisition of contextual fear memory that we have observed with weaker US [5]. We conclude that SST becomes particularly relevant for conditioning under mildly aversive conditions and deficits of SST^-/-^ mice can be overcome by moderate or strong fear conditioning training. At the same time our data demonstrate that SST mediates the modulatory influence of circadian rhythm on innate anxiety. Bearing in mind that innate anxiety and conditioned fear are mediated by overlapping, but not identical brain circuits [25] it is interesting whether the involvement of the SST system in innate fear may be similarly dependent on stimulus aversiveness. In fact, a recent study sheds light on this question by demonstrating an activation of SST-positive interneurons in the BLA after mild behavioral stress in the elevated plus maze test and a suppression in the BLA and CeA after exposure to ferret odor that elicits a strong fear response [26]. In the same study interneurons that express NPY were found to be activated after strong stimuli within different subnuclei of the amygdala. NPY is co-expressed with SST in interneuron populations of the amygdala [7] and also mediates anxiolytic responses via different receptors in the BLA [27], decreasing cell excitability of this structure [28]. Thus it is interesting that NPY showed a significant circadian regulation of gene expression similar to SST in the BLA, raising the possibility that the two co-expressed neuropeptides may complement each other functionally under differently aversive conditions. 

Future studies will have to further specify the roles of these and other peptides in the fine regulation of the anxiety versus the fear network. The identification of interacting and compensatory factors in SST^-/-^ mice may provide important clues to these functions. One candidate is structurally and functional closely related neuropeptide cortistatin, which however appears not to be altered in expression in SST^-/-^ mice. In contrast, increased levels of the SST R2 receptor subtype have been described in the hypothalamus and the hippocampus of these animals [29,30], that functionally compensate, at least in part, for SST deficiency. However, binding of SST is not increased in the BLA of SST^-/-^ mice, but SST R2 expression has not yet been studied in detail in this region [31].

Neuropeptide systems are likely to interact with other circadian modulators in the control of fear circuits in the amygdala. Plasma levels of corticosterone, chronic or acute injections of which increase anxiety-like behavior [32,33], display a circadian oscillation with high levels at the beginning of the dark phase and low concentrations towards the second half of the dark phase [34]. Moreover, expression of the clock gene Per2 is modulated in a circadian fashion within in the amygdala, the bed nucleus of the stria terminalis and the hippocampus [13,14]. However, this regulation is not uniform as, e.g., circadian expression of Per2 in the BLA and CeA shows opposing rhythms. Moreover, only the latter of these is sensitive to andrenalectomy [12]. This suggests that circadian fluctuations of corticosterone exert strong influence on CeA and other parts of the fear and anxiety circuitry [13,22,35], but spare regulation of Per2 and its targets in the BLA. In the current study, we confirmed the circadian regulation of Per2 expression in the BLA and its significant increase during the second half of the dark phase. SST, GAD65 and NPY mRNA levels correlated well with the circadian expression pattern of Per2 in the BLA, but no significant correlation was observed for Gad67 and cholecystokinin (CCK). 

Normally, a “time-stamping” of episodic memory [36] occurs to set a temporal context in memory formation and allows for improved retrieval of information at the same time of the day as the initial training [37]. However, SST^-/-^ mice as well as Per2^-/-^ mice [14] display no deficits in auditory-cued fear conditioning. Circadian effects on behavioral tests in rodents show considerable interaction with factors like genetic background and sex [15,21] or illumination conditions during testing [38,39], suggesting a complex interplay of various factors and brain regions. Moreover, the relevance of time stamping can be expected to decline in the case of robust and implicit memories. Nevertheless we have recently observed in GAD65^-/-^ mice disturbed state-specific fear memory expression of which was regulated in dependence of the circadian rhythm (Bergado-Acosta, Müller and Stork, unpublished). Our current data suggest that in contrast to GAD65, SST is not required to generate state-specific fear memory, but contributes to circadian fluctuation in innate anxiety and modulates conditioning to mildly aversive stimuli in mice.
